# Comparison of sonoelastographic values of breast tissue with mammographically and ultrasonically assessed density: a cross-sectional study

**DOI:** 10.3325/cmj.2020.61.223

**Published:** 2020-06

**Authors:** Martina Džoić Dominković, Gordana Ivanac, Boris Brkljačić

**Affiliations:** 1 Department of Radiology, General Hospital Orašje, Orašje, Bosnia and Herzegovina; 2 Department of Diagnostic and Interventional Radiology, University Hospital Dubrava, Zagreb, Croatia; 3 University of Zagreb School of Medicine, Zagreb, Croatia

## Abstract

**Aim:**

To determine the relationship between breast stiffness assessed with sonoelastography (elasticity) and breast tissue density assessed with mammography (MG) and ultrasound (US).

**Methods:**

This cross-sectional study involved 100 women who underwent MG, gray-scale US, and shear-wave sonoelastography during 2013. Mammographic density was categorized into four groups and sonographic density into three groups according to Breast Imaging-Reporting and Data System criteria. The stiffness of breast parenchymal and adipose tissue in all breast quadrants was quantified by shear-wave sonoelastography. Mean elastographic estimates were compared with MG- and US-derived density estimates.

**Results:**

Parenchymal and adipose tissue elasticity positively correlated with MG- and US-derived breast density (for parenchyma: for MG Kendall’s tau_b 0.522; Jonckheere-Terpstra test *P* < 0.001 and for US Kendall’s tau_b 0.533; Jonckheere-Terpstra test *P* < 0.001); the higher was the breast density on MG and US, the higher was the elastographic stiffness.

**Conclusion:**

Sonoelastographic breast stiffness strongly positively correlated with breast density. Thus, sonoelastography may have a potential for estimating the breast cancer risk, which allows a novel application of this technique in routine clinical practice.

Breast tissue composition is determined by the relative proportions of glandular, fibrous, and adipose tissue. Glandular tissue has long been considered a key factor in breast physiology and pathology, but recent studies have also investigated the roles of adipose and fibrous tissue (1). Mammographic breast density is expressed as the percentage of the mammogram occupied by radiologically dense (glandular and fibrous) tissue (2). Breast density positively correlates with the occurrence of proliferative breast diseases without atypia (3).

The Breast Imaging-Reporting and Data System (BI-RADS), designed and developed by the American College of Radiology (ACR), has been employed in the United States since 1992 and provides a standardized approach for interpreting medical imaging examinations for breast diseases. In the BI-RADS edition 2003, breast composition was classified based on the overall density as follows: ACR category 1 (<25% fibroglandular tissue), category 2 (25%-50%), category 3 (50%-75%), and category 4 (>75%). Since the chance that a mass can be obscured by fibroglandular tissue is a better indicator of breast cancer risk than the percentage of breast density, BI-RADS 2013 stopped using percentages and classified breast composition as follows: A – the breasts are almost entirely fatty, which increases the mammographic sensitivity; B – there are scattered areas of fibroglandular density (density describes the degree of x-ray attenuation of breast tissue but not discrete mammographic findings); C – the breasts are heterogeneously dense, which can obscure small masses; D – the breasts are extremely dense, which lowers the mammographic sensitivity (4) (Figure 1A).

**Figure 1:**
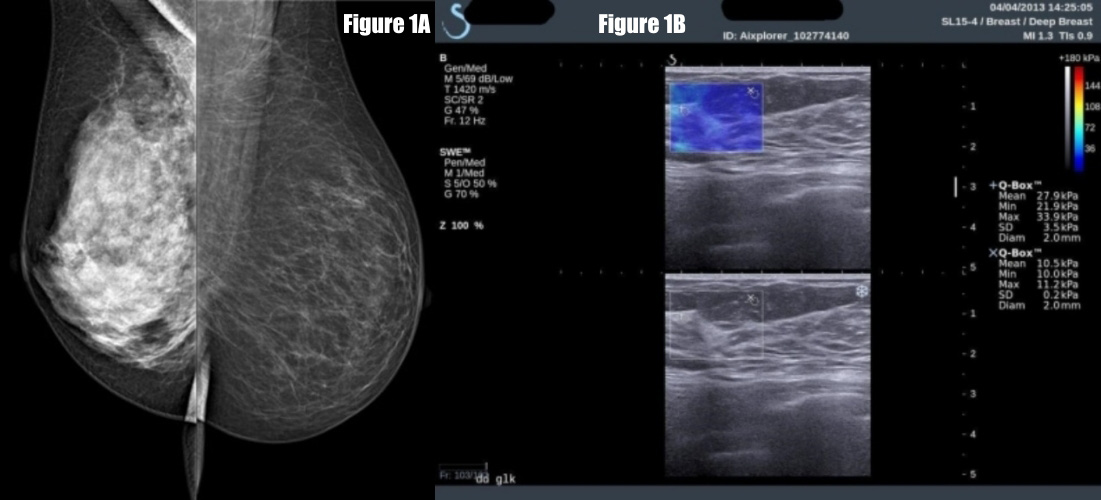
(**A**) Different breast densities on mammography: left – extremely dense breast; right – entirely fatty breast. (**B**) Sonoelastographic measurement showing heterogeneous breast density. The region of interest is placed in the representative area of parenchymal and adipose tissue. Ultrasound device shows elasticity values in color and kilopascals.

Breasts composition and structure evaluated on gray-scale (B-mode) ultrasound can be classified into three categories according to the ACR BI-RADS criteria: homogeneously fatty; homogeneously fibroglandular; and heterogeneous (mixed form) (4).

The mechanical properties of breast tissue, such as stiffness, and the effect of mechanical forces on the physiology of breast tissue have very seldom been examined (5). However, many types of soft tissue can have similar echogenicity while differing considerably in elastic properties (6). The evaluation of elasticity might therefore help to detect the areas of pathologically altered breast tissue (6,7). The aim of this study is to evaluate the relationship between breast stiffness assessed with sonoelastography and breast tissue density assessed with mammography (MG) and ultrasound (US). Our hypothesis was that breasts with higher mammographic density had higher sonoelastographic values.

## MATERIALS AND METHODS

### Patients

The single-center, observational, cross-sectional study involved 100 women who underwent screening or diagnostic MG and elastography in the Department of Diagnostic and Interventional Radiology, University Hospital Dubrava, Zagreb, Croatia during 2013. We allocated 25 women to each of the four groups of patients with different mammographic parenchymal density according to BI-RADS criteria to have the same number of patients in every breast density group (4) (Table 1) (Figure 1A). The examiner was not blinded to the mammographic findings when she interpreted the ultrasound, and *vice versa* but she first analyzed mammographic findings and then the ultrasound findings. This study was approved by the Institutional Review Board of University Hospital Dubrava, and all patients provided informed consent for research participation and data publication.

**Table 1:** Ultrasonically and mammographically assessed breast tissue densities and elastographic values of parenchymal and adipose tissue

Density level	Number of patients	Elastographic values (median and range), kPa	
		parenchymal tissue	adipose tissue
**Mammography groups**			
entirely fatty breasts	25	37.6 (32.7-43.5)	21.8 (16.7-23.7)
breasts with scattered areas of fibroglandular density	25	55.3 (42.2-73.8)	24.6 (21.3-32.3)
heterogeneously dense breasts	25	66.3 (52.9-84.7)	27.3 (20.9-32.1)
extremely dense breasts	25	83.2 (67.9-101.5)	29.5 (23.2-38.2)
**Ultrasound groups**			
homogeneously fatty breasts	25	37.6 (31.4-44.6)	21.9 (16.6-23.9)
heterogeneous breasts (mixed form)	50	63.0 (44.7-79.4)	26.3 (20.8-32.3)
homogeneously fibroglandular breasts	25	83.2 (65.9-102.1)	29.5 (22.4-38.2)

## Methods and instruments

Screening MG was performed as part of a national mammographic screening program or, for younger women, due to a family history of breast cancer. Diagnostic MG was performed for patients experiencing a variety of symptoms. We excluded women who had undergone surgery in the thoracic area with radiation therapy including the breasts and those already known to have breast tumors. MG was performed using a full-flat panel digital mammography system (Mammomat Novation DR, Siemens, Erlangen, Germany). Standard mediolateral oblique and craniocaudal projections were performed and reviewed at dedicated workstation. All women underwent US examination on an Aixplorer scanner (Supersonic Imagine, Aix en Provence, France, software version 6.2.23751, product version 6.2.0). Based on the appearance of the breast tissue on gray-scale US, women were divided into three groups as per ACR BI-RADS criteria (4): homogeneously fatty breasts (25 women), heterogeneous breasts (50 women), and homogeneously fibroglandular breasts (25 women) (Table 1).

Shear-wave elastography (SWE) examination was performed on an Aixplorer scanner with a high-frequency 4-15 MHz linear transducer. SWE without manual compression was added to routine US examination for the purposes of the study. Breast tissue stiffness (parenchymal and fat tissue) was measured with built-in quantification region of interest (ROI, Q-Box; SuperSonic Imagine). The Q-Box provides sonoelastographic measurements in kilopascals (kPa) in a ROI. We used a 2-mm ROI for all measurements. The first ROI was placed in the parenchymal breast tissue and the second ROI of the same size was placed in the fatty breast tissue. Ultrafast imaging captures shear waves, and Aixplorer quantifies propagation speed using colors – from dark blue (lowest stiffness) to red (highest stiffness), which results in a real-time elastographic color map. It also shows elasticity values in kilopascals: at just over 0 to ≥180kPa (7.7 m/s) (Figure 1B). Every breast was divided into four quadrants, and one measurement of glandular parenchyma elasticity and one measurement of fat tissue elasticity in each quadrant was performed, ie, eight measurements per breast. The ultrasound probe was placed in the middle of each quadrant (transversal planes). All elastographic measurements were made with the same device preset, “penetration mode.” At each ROI positioning and sonoelastographic measurement, maximum E value, mean E value, and minimum E value in kilopascals are displayed on the ultrasound scanner screen. Mean E values were recorded for parenchymal tissue and fat tissue in every quadrant, and the average value of four E mean measurements in each breast was used for comparisons. All measurements (MG, US, and sonoelastography) were performed by one radiologist with more than 5 years of experience in breast radiology.

### Statistical analysis

Descriptive statistics was used to summarize the data. Categorical and nominal values are expressed as frequencies and percentages, and continuous data are presented as median and range. The Jonckheere-Terpstra test was used to determine if sonoelastography values significantly increased with the increase in mammographic and ultrasonic categories, and the correlation between the variables was assessed with Kendal's tau_b correlation coefficient. The level of significance was set at 0.05. The analysis was conducted with IBM SPSS, version 25 (IBM, Armonk, NY, USA) and StatsDirect, version 3.1.12 (StatsDirect Ltd, Birkenhead, UK).

## RESULTS

**Figure 2:**
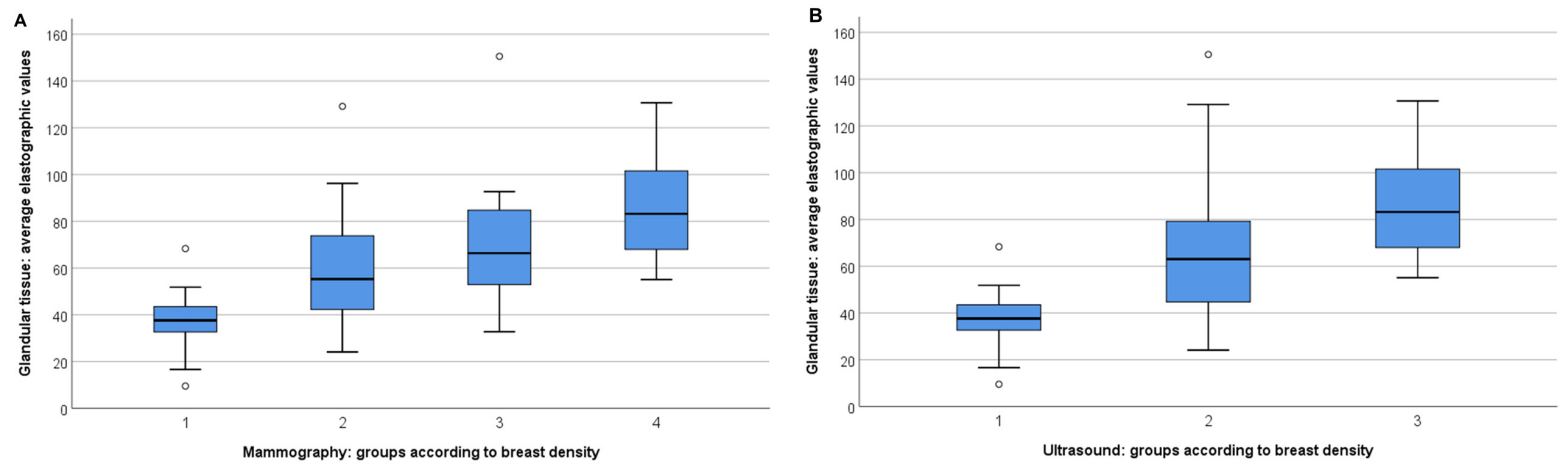
(**A**) The association between the average elasticity values of parenchymal tissue and estimated mammographic density categorized according to American College of Radiology Breast Imaging-Reporting and Data System criteria. (**B**) The association between average elasticity values of parenchymal breast tissue and estimated ultrasound density categorized according to American College of Radiology Breast Imaging-Reporting and Data System criteria. Elasticity values are expressed as kilopascals.

**Figure 3:**
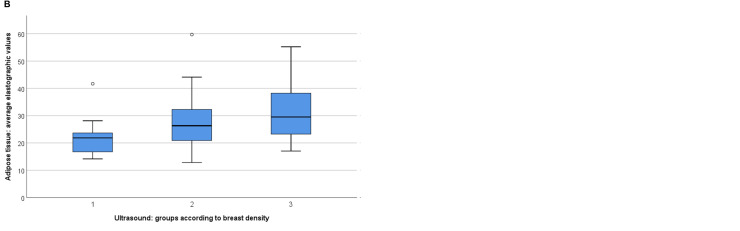
(**A**) The association between average elasticity values of adipose tissue and estimated mammographic density categorized according to American College of Radiology Breast Imaging-Reporting and Data System criteria. (**B**) The association between average elasticity values of adipose breast tissue and estimated ultrasound density categorized according to American College of Radiology Breast Imaging-Reporting and Data System criteria. Elasticity values are expressed as kilopascals.

The mean age was 51.6 years (standard deviation 9.63; minimum age was 35 and maximum 73 years). The average sonoelastographic measurement values obtained in MG and US categories of breast tissue density are shown in Table 1. Average sonoelastographic values of parenchymal tissue positively correlated with mammographic (Kendall’s tau_b 0.522; Jonckheere-Terpstra test *P* < 0.001) and ultrasonic (Kendall’s tau_b 0.533; Jonckheere-Terpstra Test *P* < 0.001) parenchymal density categories (Figure 2). Average sonoelastographic values of adipose tissue positively correlated with mammographic (Kendall’s tau_b 0.280; Jonckheere-Terpstra test *P* < 0.001) (Figure 3A) and ultrasonic (Kendall’s tau_b 0.304; Jonckheere-Terpstra test *P* < 0.001) density of adipose tissue (Figure 3B). Thus, the higher were the mammographic and ultrasonic categories of breast density, the higher was the stiffness of both parenchymal and adipose breast tissue assessed with sonoelastography (Figure 2 and 3).

## DISCUSSION

This study showed that both parenchymal and adipose breast tissue stiffness assessed with sonoelastography positively correlated with mammographic and ultrasonic breast density categories. According to McCormack and dos Santos Silva (8), mammographically estimated breast tissue density was directly associated with an increased risk of breast cancer. The relative risk of breast cancer increased from 1.8 for adipose breasts to 4.6 for dense breasts as defined with BI-RADS criteria (8). The mechanisms behind the association between increased breast cancer risk and breast density are not fully understood (9). Breast cancer arises from the epithelial cells of glandular epithelial tissue, and density represents the proportion of glandular and fibrous tissue in a breast. Therefore, a higher amount of parenchyma means a greater number of cells that are at risk of carcinogenesis and epithelial proliferation. The mammographic density percentage decreases with age, which seems paradoxical given that breast cancer incidence increases with age (9-11). Women with denser breasts have a higher risk of developing breast cancer and other proliferative lesions that can be precursors to breast cancer (12-21). The women whose breast density category changed from a higher to a lower had a lower risk of developing breast cancer (22).

Mammographic breast density assessment has a few limitations. This method does not take into account breast thickness, and therefore the assessment is based only on the projected area rather than the tissue volume. Computer-assisted methods of measuring breast density only differentiate between dense and non-dense breasts, not recognizing any transitional forms (23). Variations in the current or voltage used in generating the image are also not considered. An experienced radiologist is needed as an observer, which leads to subjective measurements. The potential sources of measurement error can weaken the levels of association between mammographic density percentage and other risk factors for developing breast cancer. Furthermore, the radiation exposure limits the possibility of repeated measurements and, except for patients with clinical indication, precludes the use of mammography in young women. There are also other possibilities for measuring breast density: magnetic resonance and ultrasound tomography (23). Mammographically estimated breast tissue stiffness is associated with the risk of breast cancer (24), and mechanical imaging added to mammography breast screening can detect increased pressure in breasts with benign and malignant lesions (25).

This study found a good correlation between sonoelastographic breast stiffness and breast density. Two studies that compared similar data yielded conflicting results (26,27). One study using virtual touch tissue imaging quantification to ultrasonically measure tissue stiffness found no significant difference between ACR breast density categories (26). They divided breasts in more and less dense breasts (ACR 1 + 2 and ACR 3 + 4). The measurement process was not explained in detail, and sonoelastography is prone to different results if different measurement techniques are used (26). The second study found that B-mode ultrasound and elastography successfully predicted mammographic density percentage (27).

The main limitations of this study are a small sample size and the lack of assessment of other factors that influence mammographic density. It would be interesting to see how much elastic characteristics of breast tissue depend on physiological changes in breast tissue (age, day of menstrual cycle, hormonal status, parity, duration of lactation, and others). These factors were not considered because the aim of the study was to assess only the correlation between mammographic density and sonoelastographic stiffness and not why some breasts are denser than others. A further limitation of this study is the lack of standardization of sonoelastographic measurement. A more standardized measurement could decrease the method’s subjectivity and influence compression during examination.

We demonstrated an association between mammographic density percentage and sonoelastographic stiffness, which means that women could be stratified according to breast cancer risk by using sonoelastography, a fast, cheap and repeatable method that does not expose women to ionizing radiation and can therefore easily be performed in younger women. In addition, SWE measurements provide quantitative data in kilopascals, while MG provides only qualitative and semiquantitative data. It would be interesting to determine whether, like higher mammographic density, higher sonoelastographic stiffness itself is a risk factor for breast cancer development, and whether mammographic density and sonoelastographic stiffness are influenced by similar factors. If this is so, stiffness and density might be considered roughly equivalent categories evaluated with different imaging methods.
